# Cooperation between chemotherapy and immune checkpoint blockade to enhance anti-tumour T cell immunity in oesophageal adenocarcinoma

**DOI:** 10.1016/j.tranon.2022.101406

**Published:** 2022-03-30

**Authors:** Maria Davern, Noel E. Donlon, Fiona O’ Connell, Andrew D. Sheppard, Conall Hayes, Ross King, Hugo Temperley, Christine Butler, Anshul Bhardwaj, Jenny Moore, Dara Bracken-Clarke, Claire Donohoe, Narayanasamy Ravi, John V. Reynolds, Stephen G. Maher, Melissa J. Conroy, Joanne Lysaght

**Affiliations:** a Cancer Immunology and Immunotherapy Group, Department of Surgery, Trinity St. James's Cancer Institute, Trinity Translational Medicine Institute, St. James's Hospital campus, Dublin 8, Ireland; b Department of Surgery, Trinity St. James's Cancer Institute, Trinity Translational Medicine Institute, St. James's Hospital, Trinity College Dublin, Dublin, Ireland

**Keywords:** PD-1, A2aR, CTLA-4, FLOT regimen, CROSS regimen

## Abstract

Response rates to immune checkpoint blockade (ICB) remain low in oesophageal adenocarcinoma (OAC). Combining ICB with immunostimulatory chemotherapies to boost response rates is an attractive approach for converting ‘cold’ tumours into ‘hot’ tumours. This study profiled immune checkpoint (IC) expression on circulating and tumour-infiltrating T cells in OAC patients and correlated these findings with clinical characteristics. The effect of first-line chemotherapy regimens (FLOT and CROSS) on anti-tumour T cell immunity was assessed to help guide design of ICB and chemotherapy combinations in the first-line setting. The ability of ICB to enhance lymphocyte-mediated cytolysis of OAC cells in the absence and presence of post-FLOT and post-CROSS chemotherapy tumour cell secretome was assessed by a CCK-8 assay. Expression of ICs on T cells positively correlated with higher grade tumours and a subsequent poor response to neoadjuvant treatment. First-line chemotherapy regimens substantially altered IC expression profiles of T cells increasing PD-1, A2aR, KLRG-1, PD-L1, PD-L2 and CD160 and decreasing TIM-3 and LAG-3. In addition, pro-inflammatory T cell cytokine profiles were enhanced by first-line chemotherapy regimens. T cell activation status was significantly altered; both chemotherapy regimens upregulated co-stimulatory markers ICOS and CD69 yet downregulated co-stimulatory marker CD27. However, ICB attenuated chemotherapy-induced downregulation of CD27 on T cells and promoted differentiation of effector memory T cells into a terminally differentiated state. Importantly, dual nivolumab-ipilimumab treatment increased lymphocyte-mediated cytolysis of OAC cells, an effect further enhanced in the presence of post-FLOT tumour cell secretome. These findings justify a rationale to administer ICBs concurrently with first-line chemotherapies.

## Highlights

• Immune checkpoints correlate with higher grade tumours and poor treatment response.
• First-line chemotherapies stimulate anti-tumour T cell immunity.
• Dual nivolumab-ipilimumab enhances lymphocyte-mediated killing of tumour cells.
• Lymphocyte-mediated killing of tumour cells is enhanced by FLOT chemotherapy.

## Graphical abstract

Study schematic **-** (A) Immune checkpoint (IC) expression profiles of T cells in tumour biopsies and whole blood from OAC patients was assessed by flow cytometry and correlated with clinical demographics. (B) The effect of first-line chemotherapy regimens on anti-tumour T cell phenotypes was next assessed using flow cytometry. PBMCs were isolated from age-matched healthy donor and OAC donor blood and expanded *ex vivo* for 72 h using anti-CD3/28 + IL-2 T cell activation protocol followed by an additional 48 h treatment with vehicle, FLOT or CROSS chemotherapy (CT) regimens in the absence and presence of immune checkpoint blockade (ICB). (C) The ability of ICB to boost the cytolytic effector function of OAC donor lymphocytes was next assessed in the absence and presence of post-vehicle/FLOT/CROSS CT tumour cell secretome to determine if the chemotherapy altered tumour microenvironment enhanced lymphocyte-mediated killing of tumour cells. Collection of post-chemotherapy tumour cell secretome: OE33 cells were treated for 48 h with vehicle/FLOT/CROSS CT and washed twice with PBS. Tumour cell secretome was then harvested an additional 48 h later. Key Findings – (D) ICs were upregulated on tumour-infiltrating T cells. CTLA-4, TIM-3 and ICOS were decreased 6 weeks post-FLOT or CROSS chemoradiotherapy treatment on tumour-infiltrating T cells. (E) IC expression correlated with more advanced staged tumours, nodal metastasis and poor response to treatment. (F) The direct 48 h effects of FLOT and CROSS CT treatment revealed that these regimens upregulated a range of ICs and downregulated TIM-3 and LAG-3 on T cells *ex vivo*. Co-stimulatory markers were differentially affected, ICOS and CD69 were upregulated however CD27 was downregulated on T cells. Anti-tumour T cell cytokine profiles were also enhanced. (G) ICB attenuated the chemotherapy-mediated downregulation of CD27. Dual nivolumab and ipilimumab treatment increased lymphocyte-mediated killing of tumour cells which was further enhanced in the presence of post-FLOT tumour cell secretome suggesting the potential of immunostimulatory synergism between first-line OAC chemotherapy regimens and ICB.

**Figure:**
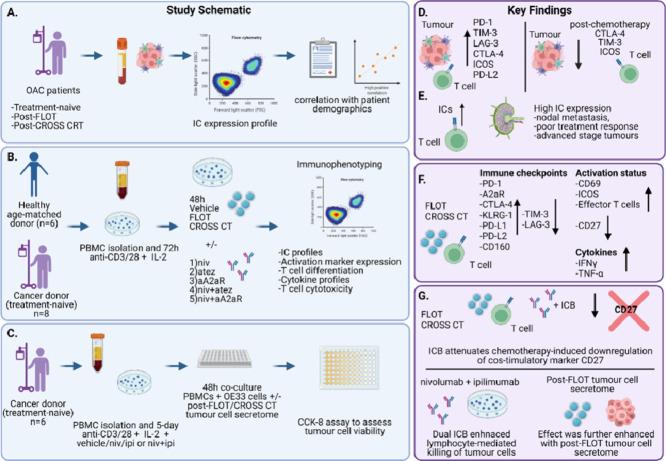

## Introduction

Targeting inhibitory immune checkpoints (ICs) is an attractive therapeutic strategy to reinvigorate exhausted anti-tumour immunity in oeosphageal adenocarcinoma (OAC) [1]. The current standard of care for resectable OAC includes the peri-operative FLOT chemotherapy-based regimen [2]. The FLOT regimen includes 5-fluorouracil (5-FU), leucovorin, oxaliplatin and a taxane (such as the anti-microtubule agent docetaxel) before (neoadjuvant) and after surgery (adjuvant). Multimodal chemoradiotherapy is also an option for OAC patients and includes the CROSS regimen (paclitaxel and carboplatin with a cumulative radiation dose of 41.4Gy over 23 fractions) followed by surgery [3]. However, a significant proportion of OAC patients fail to derive a curative response from current standards of care, with only approximately 30% of patients achieving a complete pathological response [4]. Immune checkpoint blockers (ICBs) are an immunotherapeutic option for OAC patients and have already exhibited clinical efficacy in a wide range of cancer types [1]. Immunotherapy is now considered by many as the fifth pillar of cancer therapy along with surgery, chemotherapy, radiotherapy and molecular targeted therapies [5].

Immune checkpoint (IC) pathways control the magnitude and duration of the immune response, preventing overactivation of the immune system, which could lead to the development of autoimmunity [6]. ICBs block IC pathways, reinvigorating anti-tumour immunity [5]. Single agent pembrolizumab (Keytruda), an anti-PD-1 monoclonal antibody, was FDA-approved for the treatment of advanced or recurrent oesophagogastric cancers in the third-line setting for tumours expressing PD-L1 (combined positive score (CPS) ≥1) [7]. In 2021, nivolumab (Opdivo) was FDA approved for patients with completely resected oesophageal or gastroesophageal junction cancer with residual pathologic disease who have received neoadjuvant chemoradiotherapy based on findings from the CHECKMATE-577 trial (NCT02743494).

To date the majority of clinical trials in all cancers including OAC have largely focussed on testing the efficacy of blocking PD-1 and CTLA-4 IC pathways, despite the vast array of potentially targetable ICs expressed on the surfaces of T cells [8], [9], [10], [11].

Novel ICs which represent targetable therapeutic options for OAC patients, to be given alone or in combination with PD-1 or CTLA-4 ICB, include lymphocyte activation gene-3 (LAG-3), T cell immunoglobulin-mucin domain-3 (TIM-3), T cell immunoglobulin and ITIM domain (TIGIT), adenosine A2a receptor (A2aR) [5] and CD160 (ligand for herpes virus entry mediator) [5]. Despite belonging to the same class of receptors as PD-1 and CTLA-4, the ICs TIM-3, TIGIT and LAG-3 exhibit unique functions, especially at tissue sites where they regulate distinct aspects of immunity [12].

Interestingly, recent studies have demonstrated that combining immune checkpoint blockers (ICB) with the standard of care chemotherapy regimens in oesophageal adenocarcinoma (OAC) patients can boost clinical outcomes [13]. ICBs are thought to be largely ineffective in non-immunogenic ‘cold’ tumours, where there is an absence of pre-existing anti-tumour immunity and therefore no immune response to reinvigorate [14]. However, chemotherapies are emerging as a valuable tool to convert ‘cold’ tumours to ‘hot’ tumours through different mechanisms. For example, chemotherapy-induced DNA damage in cancer cells can generate neoantigens, which then activate anti-tumour specific T cell responses. This is an attractive strategy to sensitise TMB-low tumours to ICBs [15]. In addition, immunostimulatory chemotherapies induce immunogenic cell death via the release of damage-associated molecular patterns into the extracellular tumour microenvironemnt [16]. Damage-associated molecular patterns induce maturation and activation of dendritic cells and subsequent activation and mobilisation of anti-tumour T cells to the tumour site [17].

To support the synergy between chemotherapy and ICB combinations in OAC the phase III CheckMate 649 trial demonstrated that combining nivolumab with first-line chemotherapy (FOLFOX and XELOX) in previously untreated oesophagogastric junctional (OGJ) cancer patients (*n* = 1581), significantly improved overall survival in patients with a PD-L1 combined positive score of 5 or greater (14.4 months (nivolumab + chemotherapy arm) vs. 11.1 months (chemotherapy arm)) [13]. Furthermore, the nivolumab + chemotherapy arm also reduced the risk of death by 29% (HR, 0.71; 98.4% CI, 0.59–0.86; *p* < 0.0001) [13]. The findings from this trial highlight the potential therapeutic synergy that can be exploited between chemotherapy and ICB.

However, the effects of chemotherapy on IC expression profiles in the context of OAC remain unknown, as are the direct effects of first-line chemotherapy regimens on anti-tumour T cell responses in OAC. This study aims to address these important gaps in research knowledge by profiling IC expression in OAC patients in the neoadjuvant and adjuvant setting. The direct effects of first-line chemotherapy regimens FLOT and CROSS on T cell cytokine profiles and anti-tumour T cell responses are also investigated. These findings may help inform the selection of appropriate ICs to target in OAC and the sequenced timing of ICB with current standards of care.

## Methods

### Ethical approval

All samples were collected with prior informed written consent for sample and data acquisition from patients attending St. James's Hospital or from healthy donors. This study was carried out in accordance with the World Medical Association's Declaration of Helsinki guidelines on medical research involving human subjects. Patients provided informed consent for sample and data acquisition, and the study received full ethical approval from the St. James's Hospital/AMNCH Ethical Review Board. Patient samples were pseudonymised in line with GDPR and data protection policies to protect the privacy and rights of the patients.

### Specimen collection

All patients involved in this study were enroled from 2018–2020. Treatment-naïve tumour tissue biopsies were obtained from OAC patients undergoing endoscopy at St. James's Hospital at time of diagnosis prior to initiation of chemotherapy or radiotherapy. Post-FLOT chemotherapy-treated and post-CROSS chemoradiotherapy-treated OAC tumour tissue biopsies were obtained approximately 6 weeks post-treatment at time of surgical tumour resection. The group consisted of 16 males and 6 females, with an average age of 66.4 years. The patient demographics are detailed in Table 1.

**Table 1:** Patient demographic table.

Table I	
Patient Demographic Table.	
Age (years)	66.4
Sex ratio (M:F)	16:6
Diagnosis (no. patients)	
OGJ	21
OAC	2
Clinical tumour stage (no. patients)	
T0	0
T1	1
T2	6
T3	13
T4	0
Clinical nodal status^a^ (no. patients)	
Positive	10
Negative	10

### OAC Tumour Tissue Digestion

Biopsies were enzymatically digested to perform OAC cell phenotyping. Briefly, tissue was minced using a scalpel and digested in collagenase solution (2 mg/ml of collagenase type IV (Sigma) in Hanks Balanced Salt Solution (GE healthcare) supplemented with 4% (v/v) foetal bovine serum) at 37 °C and 1500 rpm on an orbital shaker. Tissue was filtered and washed with FACs buffer (PBS containing 1% foetal bovine serum and 0.01% sodium azide). Cells were then stained for flow cytometry.

### Cell culture

Age-matched healthy donor PBMCs (*n* = 6) or treatment-naïve OAC donor PBMCs (*n* = 8) were isolated from whole blood using density gradient centrifugation and expanded using a T cell activation protocol which included plate bound anti-CD3 (10 μg/ml, Biolegend, USA), anti-CD28 (10 μg/ml, Ancell, USA) and recombinant human IL-2 (Immunotools, Germany) for 3 days followed by 48 h treatment with an IC_50_ dose of a combination of chemotherapies that comprise the FLOT regimen (5-FU 0.01 µM, oxaliplatin 0.01 µM and docetaxel 0.00001 µM) or the CROSS chemotherapy (CT) regimen (paclitaxel 0.0001 µM and carboplatin 50 µM) or a vehicle control (0.0001% DMSO, 0.001% H_2_O) in the absence and presence of nivolumab (10 μg/ml), atezolizumab (10 μg/ml), A2aR antagonist (3 μM), dual nivolumab-atezolizumab (10 μg/ml and 10 μg/ml, respectively), or dual nivolumab-A2aR antagonism (10 μg/ml and 3 μM, respectively). PBMCs were grown in complete RPMI 1640 medium with 2 mM L-glutamine (Gibco) (supplemented with 1% (v/v) penicillin-streptomycin (50 U/ml penicillin 100 μg/ml streptomycin) and 10% (v/v) foetal bovine serum (Gibco)) and maintained in a humidified chamber at 37 °C 5% CO_2_. OE33 cells were purchased from European Collection of Cell Cultures. OE33 cells were grown in complete RPMI 1640 medium with 2 mM L-glutamine and maintained in a humidified chamber at 37 °C 5% CO_2_. Cell lines were tested regularly to ensure mycoplasma negativity.

### Whole blood staining

Fluorochrome-conjugated antibodies were added to 100 µl blood at pre-optimized concentrations and incubated for 15 min at room temperature in the dark. Red cells were lysed using red blood cell lysing solution (Biolegend, USA), according to manufacturer's recommendations and cells were washed twice with FACs buffer. Cells were fixed for 15 min in 1% paraformaldehyde solution (Santa Cruz Biotechnology, USA) prior to flow cytometric analysis.

### Flow cytometry staining

Whole blood, tumour tissue biopsies, healthy donor PBMCs or OAC donor PBMCs were stained with zombie aqua viability (Biolegend, USA) dye. Antibodies used for staining included ICOS-PE-efluor610, LAG-3-FITC, CD160-PerCPCy5.5, PE-1-PE/Cy7, TIGIT-PE/Cy7, CD45RA-PE/Cy7, CD45RO-BV510, CD3-APC, CD3-PerCP, CD4-BV510, CD4-APC (Biolegend, USA), CD69-PE, CD62L-FITC, CD8-BV421 (BD Biosciences, USA), CD27-APEefluor780 (eBioscience, USA), TIM-3-AF647, CTLA-4-PE/Cy5, KLRG-1-APC, PD-L1-FITC, PD-L2-PE (BD Bioscience, USA), A2aR-PE (Bio-techne, USA). PBMCs were resuspended in FACs buffer and acquired using BD FACs CANTO II (BD Biosciences) using Diva software and analysed using FlowJo v10 software (TreeStar Inc.).

For intracellular cytokine staining PBMCs were treated with PMA (10 ng/ml) and ionomycin (1 µg/ml) for the last 4 h of the incubation. Anti-CD107a-PE (BD Biosciences, USA) was added during stimulation. For the last 3 h of the incubation PBMCs were treated with brefeldin A (10 µg/ml, eBiosciences). Cells were harvested, washed in FACs buffer and intracellular cytokines were assessed using a Fixation/Permeabilisation kit (BD Biosciences), as per manufacturer's recommendations. Cells were stained with cell surface antibodies (CD8-BV421, CD3-APC or CD3-PerCP, CD4-PerCP, CD4-APC or CD4-BV510 (Biolegend, USA)) washed, permeabilised, and then stained for intracellular cytokines: IFN-γ-BV510, IL-17A-FITC, Granzyme B-PE/Cy7, Perforin-FITC BV510 (Biolegend, USA) and TNF-α-APC (BD Biosciences, USA). Cells were resuspended in FACs buffer and acquired using BD FACs CANTO II (BD Biosciences).

### Generation of conditioned media

OE33 cells were seeded at a density of 1 × 10^6^ cells/flask in T25 flasks and the media was changed the following day. When the flasks reached 40–50% confluency the cells were treated with a combination of chemotherapies that comprise the FLOT or the CROSS CT regimen or a vehicle control for 48 h (0.0001% DMSO, 0.001% H_2_O). OE33 cells were treated for 48 h with vehicle (0.0001% DMSO and 0.0001% H_2_O), FLOT or CROSS CT chemotherapy regimens (IC_50_ doses as previously described in [18]), washed twice to remove the chemotherapy drugs and the OE33 conditioned media was harvested and stored at −80°C until required for experimentation.

### Cytolysis assay

Cytolysis assay was carried out as previously demonstrated in [19]. OAC PBMCs (*n* = 6) were isolated from treatment-naïve OAC blood using density gradient centrifugation and expanded using the T cell activation protocol in the absence and presence of nivolumab (10 μg/ml), ipilimumab (10 μg/ml), or dual nivolumab-ipilimumab (10 μg/ml and 10 μg/ml, respectively) for 5 days. OE33 cells were seeded at a density of 5 × 10^3^ cells/100 μl of media in a flat 96 well plate and incubated overnight at 37°C, 5% CO_2_. The media was replaced and expanded PBMCs were co-cultured with OE33 cells in an effector:target ratio of 5:1 and 10:1 for 48 h in the absence or presence of post-vehicle, post-FLOT or post-CROSS CT OE33 conditioned media (overall 1 in 2 dilution). PBMCs were also cultured alone to use as a control to account for changes in viability due to their presence in the well. OE33 cells were also cultured alone. Following a 48 h co-culture 5 ul of CCK-8 (Sigma, USA) was added to each well and the optical density at 450 nm and 650 nm (reference wavelength) was measured using the Versa Max microplate reader (Molecular Devices, Sunnyvale, CA, USA) to determine a viable cell number. Formula: (viability OE33 cell-lymphocyte co-culture-viability PBMCs alone)/ (viability untreated OE33 cells alone) x 100 = % live cells.

### Statistical analysis

Data were analysed using GraphPad Prism 9 (GraphPad Prism, San Diego, CA, USA) software and was expressed as mean ± SEM. Mann Whitney test was used to compare statistical differences between healthy donors and cancer donors. Benjamini-Hochberg was used to correct for multiple comparisons when comparing between multiple treatment groups. Statistical significance was determined as *p* ≤ 0.05. Spearman correlations were performed to analyse correlation data between clinical characteristics and flow data and visualised using the R package ‘corrplot’.

## Results

**Immune checkpoint proteins are significantly upregulated on tumour-infiltrating T cells compared with peripheral circulating T cells in OAC patients**

**Fig. 1:**
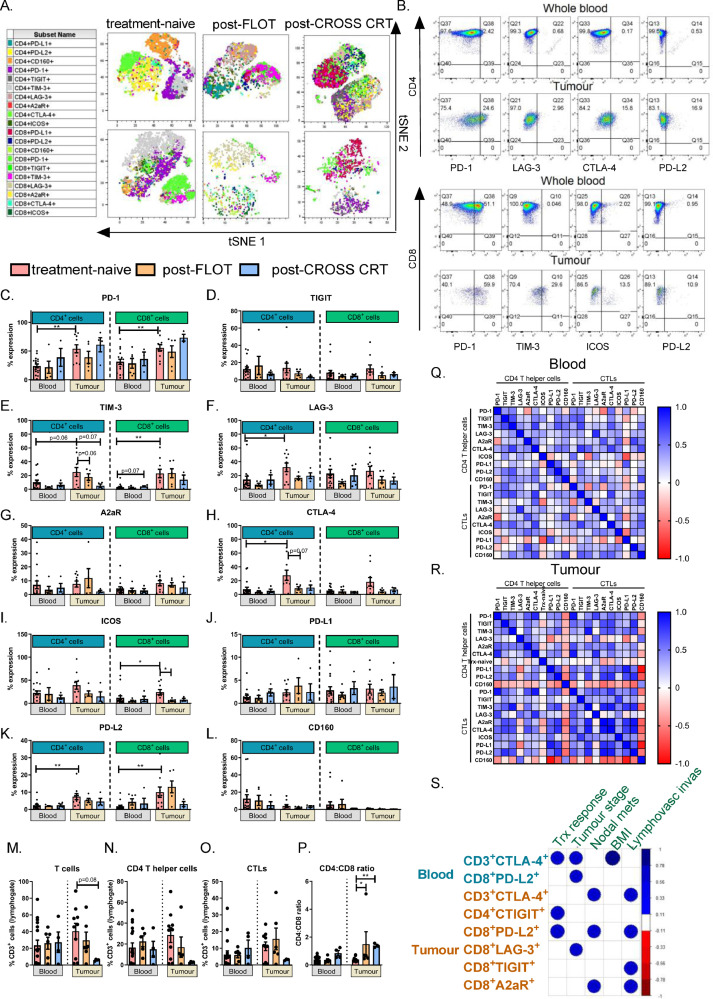
**Expression of IC receptors and ligands on the surface of circulating T cells in the periphery and tumour-infiltrating T cells in treatment-naïve, post-FLOT and post-CROSS CRT OAC patients.** (A) tSNE plots displaying spatial distribution of CD4^+^and CD8^+^cells expressing ICs in peripheral blood (top row) and infiltrating tumour tissue (bottom row) in a treatment-naïve, post-FLOT and post-CROSS CRT patient. (B) Representative dot plots depicting the ICs that were significantly upregulated on tumour-infiltrating CD4^+^ and CD8^+^ T cells compared with peripheral blood from a treatment-naïve patients. (C-L). Graphs displaying the frequency of CD4^+^ and CD8^+^ T cells expressing ICs in circulation and infiltrating tumour tissue in the treatment-naïve versus post-FLOT and post-CROSS CRT setting. Frequency of T cells (M), CD4 T helper cells (N) and CTLs (O) and the CD4:CD8 ratio assessed in whole blood and infiltrating tumour tissue in treatment-naïve versus post-FLOT and post-CROSS CRT patients. Correlation matrix for IC expression on T cells in whole blood (Q) and tumour tissue (R) in treatment-naïve setting. Corrogram displaying significant correlations between ICs and clinical data from treatment-naïve patients (S). Patient cohort includes treatment-naïve OAC patients (blood: *n* = 17 and tumour: *n* = 10), post-FLOT (blood: *n* = 6 and tumour: *n* = 6) and post-CROSS CRT (blood: *n* = 4 and tumour: *n* = 4). Mann Whitney test to compare between 2 groups and Spearman correlation used for correlative analysis *p < 0.05, **p < 0.01.

ICB to reinvigorate anti-tumour immunity has been the most successful immunotherapy in solid malignancies [20]. Blockade of the PD-1 or CTLA-4 axes in OAC and other cancer types has been the most investigated [21]. There exists a wide range of novel ICs that might also present viable therapeutic targets outside of the well-known PD-1 and CTLA-4 IC axes to stimulate anti-tumour immunity in these patients [22]. Recently a detailed single-cell analysis and transcriptional profiling of oesophageal squamous cell tumours revealed that CD8 T cells showed continuous progression from pre-exhausted to exhausted T cells [23]. Therefore, this study profiles the expression of a panel of ICs beyond the PD-1 and CTLA-4 axes including TIGIT, TIM-3, LAG-3 A2aR and ICOS and CD160, which play a prominent role in mediating T cell exhaustion on circulating and tumour-infiltrating T cells in treatment-naïve and post-treatment setting. This will shed light on the landscape of IC expression in OAC patients. tSNE plots for treatment-naive, post-FLOT and post-CROSS CRT whole blood samples (top row) and corresponding tumour biopsy tissue samples (bottom row) are showcased in Fig. 1**A.** which provide a visual presentation of the spatial distribution of IC expression profiles for CD4^+^ and CD8^+^ T cells and how they cluster together in two-dimensional plots.

Fig. 1**B.** portrays representative flow cytometry dot plots indicating the specific ICs that were significantly upregulated on tumour-infiltrating CD4^+^ and CD8^+^ T cells compared with those in peripheral blood circulation of OAC patients in the treatment-naïve setting.

PD-1 was significantly upregulated on the surface of tumour-infiltrating CD4^+^ T cells and CD8^+^ T cells compared with those in peripheral circulation in the treatment-naïve setting (*p* = 0.003 and *p* = 0.007) (Fig. 1**C.**). There appears to be an increase in Tim-3 expression on tumour-infiltrating CD4^+^ T cells and a significant increase on CD8^+^ T cells compared with circulating levels in the treatment-naïve setting (*p* = 0.06 and *p* = 0.009) (Fig. 1**E.**). TIM-3 was significantly upregulated on the surface of tumour-infiltrating CD4^+^ T cells and CD8^+^ T cells compared with circulating levels post-FLOT (*p* = 0.007) (Fig. 1**E.**). There appears to be a decrease in TIM-3 expression on the surface of tumour-infiltrating CD4^+^ T cells post-FLOT chemotherapy (*p* = 0.06) and post-CROSS chemoradiotherapy compared with the treatment-naïve setting (*p* = 0.07) (Fig. 1**E.**). In contrast, there appears to be an increase in TIM-3 expression on the surface of circulating CD8^+^ T cells post-CROSS chemoradiotherapy compared with the treatment-naïve setting (*p* = 0.07) (Fig. 1**E.**).

LAG-3 was significantly upregulated on the surface of tumour-infiltrating CD4^+^ T cells compared with those in circulation in the treatment-naïve setting and post-FLOT setting (*p* = 0.01 and *p* = 0.007) (Fig. 1**F.**).

CTLA-4 was significantly increased on the surface of tumour-infiltrating CD4^+^ T cells compared with those in circulation in the treatment-naïve setting (*p* = 0.03) (Fig. 1**H.**). There appears to be a decrease in the expression levels of CTLA-4 on the surface of tumour-infiltrating CD4^+^ T cells post-FLOT compared with the treatment-naïve setting (*p* = 0.07) (Fig. 1**H.**).

ICOS was significantly upregulated on the surface of tumour-infiltrating CD8^+^ T cells compared with those in circulation in the treatment-naïve setting (*p* = 0.01) (Fig. 1**I.**). Interestingly, there was a significant decrease in the expression of ICOS on the surface of tumour-infiltrating CD8^+^ T cells post-FLOT compared with the treatment-naïve setting (*p* = 0.01) (Fig. 1**I.**).

PD-L2 was also significantly upregulated on the surface of tumour-infiltrating CD4^+^ T cells and CD8^+^ T cells compared with the levels in circulation in the treatment-naïve setting (*p* = 0.001 and *p* = 0.001) (Fig. 1**K.**). There was no significant difference in the percentage of T cells expressing PD-L1 or CD160 in peripheral circulation compared with tumour tissue (Fig. 1**J and L.**). For the most part there was a positive correlation between the expression of ICs with other ICs on the surface of CD4 and CD8 cells (Fig. 1**Q and R.**).

In summary, inhibitory ICs TIM-3, LAG-3, CTLA-4 and PD-L2 and stimulatory IC ICOS were significantly upregulated on tumour-infiltrating T cells compared with peripheral circulating T cells in OAC patients. ICOS was significantly deceased on tumour-infiltrating CD8^+^ T cells post-FLOT treatment.

Given that the sample size for post-FLOT and post-CROSS CRT samples is low this may account for the lack of statistical significance between treatment-naïve and post-FLOT or post-CROSS CRT samples. However, there are clear trends whereby the expression of certain ICs appears to decrease post-FLOT and post-CROSS CRT, such as for CTLA-4 and ICOS. Therefore, we investigated if IC expression was altered in the treatment-naïve setting versus post-treatment (combining post-FLOT and post-CROSS CRT samples into one group). We found that we did achieve more statistical significance, for example CTLA-4 and ICOS were significantly decreased on the surface of tumour-infiltrating CD4^+^ T cells compared with the treatment-naïve setting (**Fig. S1F.** and **Fig. S1G.**). Furthermore, ICOS was significantly decreased on the surface of circulating and tumour-infiltrating CD8^+^ T cells compared with the treatment-naïve setting in OAC patients (**Fig. S1G.**).

Humphries et al., demonstrated that the levels of CD3, CD4, CD8, ICOS and PD-1 were individually predictive of better overall survival in OAC by immunohistochemistry [24]. In this study we correlated the frequency of circulating and tumour-infiltrating T cells expressing ICs with clinical characteristics within our patient cohort to help understand their potential prognostic significance. The frequency of circulating CD3^+^CTLA-4^+^ cells positively correlated with a poor pathologic response to neoadjuvant treatment determined by tumour regression grade using the Mandard scoring system (*p* = 0.04) (**Fig.1S)**. Moreover, the frequency of CD3^+^CTLA-4^+^ cells and CD8^+^PD-L2^+^ cells positively correlated with more advanced stage tumours (*p* = 0.02 and *p* = 0.04) (**Fig.1S)**.

The frequency of tumour-infiltrating CD3^+^CTLA-4^+^ cells, CD8^+^PD-L2^+^ cells and CD8^+^A2aR^+^ cells positively correlated with nodal metastasis (*p* = 0.03, *p* = 0.03 and *p* = 0.03) (**Fig.1S)**. Moreover, the frequency of tumour-infiltrating CD3^+^CTLA-4^+^, CD8^+^PD-L2^+^, CD8^+^TIGIT^+^, CD8^+^A2aR^+^ cells positively correlated with lymphovascular invasion (*p* = 0.03, *p* = 0.03 and *p* = 0.03) (**Fig.1S)**. The frequency of tumour-infiltrating CD4^+^TIGIT^+^ cells and CD8^+^PD-L2^+^cells positively correlated with a poor response to subsequent neoadjuvant treatment determined by tumour regression grade (*p* = 0.04 and *p* = 0.03) (**Fig.1S)**. The frequency of tumour-infiltrating CD8^+^LAG-3^+^cells positively correlated with more advanced stage tumours (*p* = 0.04) (**Fig.1S)**. Collectively, the frequency of tumour-infiltrating T cells expressing ICs correlated with more advanced stage tumours and subsequent poor response to neoadjuvant treatment.

**FLOT and CROSS CT treatment differentially altered IC expression on T cells from OAC patients compared to healthy donors.**

**Fig. 2:**
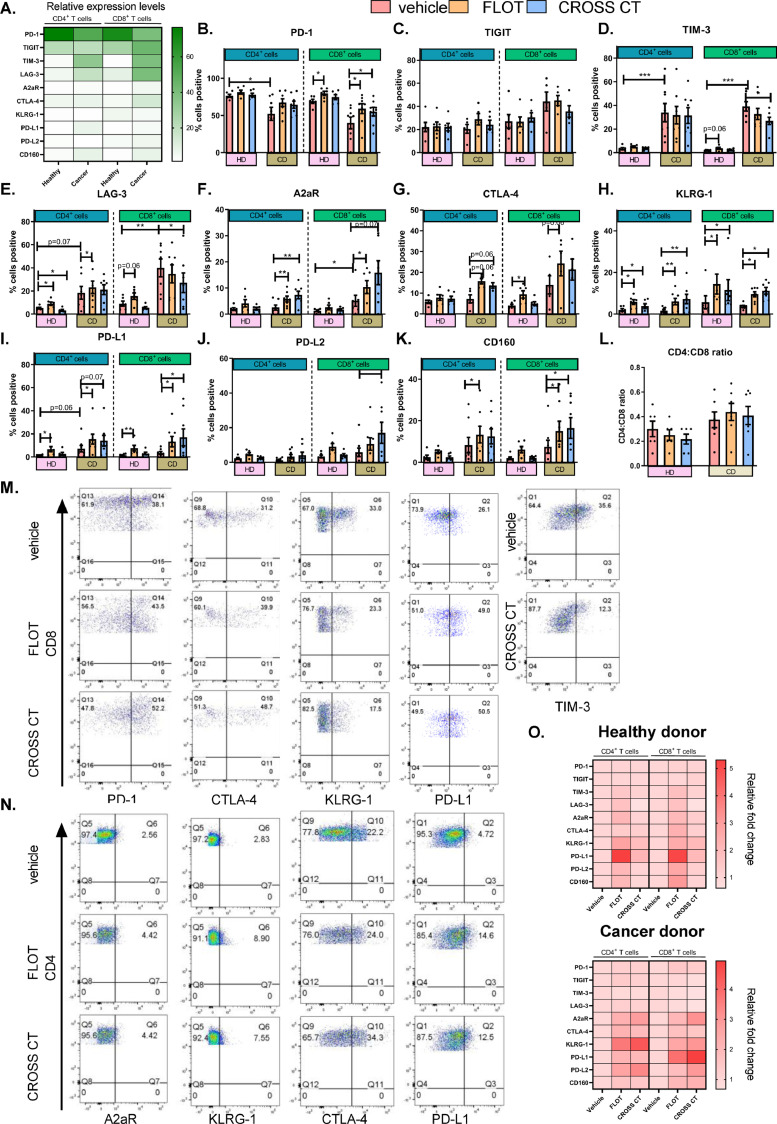
**FLOT and CROSS CT treatment significantly increases the percentage of T cells expressing PD-1, A2aR, KLRG-1 and PD-L1, while decreasing the percentage of T cells expressing TIM-3 and LAG-3.** Donor PBMCs were activated with plate bound anti-CD3, anti-CD28 and IL-2 for 72 h followed by 48 h treatment with FLOT and CROSS CT regimens. The percentage of viable CD4^+^ and CD8^+^ cells expressing IC proteins were assessed by flow cytometry. (A) Heat map summarising the expression levels of ICs on CD4 and CD8 cells from healthy donors (HDs) versus cancer donors (CDs). Graphs showing effect of vehicle, FLOT and CROSS CT on PD-1 (B), TIGIT (C), TIM-3 (D), LAG-3 (E), A2aR (F), CTLA-4 (G) and KLRG-1 (H), PD-L1 (I), PD-L2 (J) and CD160 (K) IC proteins. (L) Presents the CD4:CD8 ratio. (M) and (N) detail representative dot plots of IC expression on CD4 and CD8 cells post-vehicle, FLOT or CROSS CT from CDs. (O) Heatmaps summarising the effect of FLOT and CROSS CT on IC expression profiles of CD4 and CD8 cells as a fold change relative to the vehicle control. Healthy age-matched donors (HD) (*n* = 6) and OAC cancer donors (CD) (*n* = 8). *p < 0.05, **p < 0.01 and ***p < 0.001 Wilcoxon test to compare effect of treatments within HDs and CDs, and Mann Whitney to compare between HDs vs. CDs.

We have previously demonstrated that first-line chemotherapy regimens (FLOT and CROSS CT) significantly altered the expression profile of ICs on the surface of OAC cells [18]. However, the direct effects of FLOT and CROSS CT on IC expression profiles on T cells from OAC patients remains unknown. Therefore, to help guide the selection of the most appropriate ICs to target in combination with first-line chemotherapies in OAC, non-cancer age-matched healthy donor (HD) PBMCs and OAC cancer donor (CD) PBMCs were treated *ex vivo* with FLOT or CROSS CT and IC expression was profiled following 48 h treatment (Fig. 2**.**). Healthy donors were age-matched however, they were not sex-matched which is a potential limitation of this data.

Not surprisingly, we observed significant differences in IC expression levels on CD4^+^ and CD8^+^ T cells between HDs and CDs. The heat map in Fig. 2**A.** visually illustrates the relative expression levels of ICs on the surface of CD4^+^ and CD8^+^ T cells for both HDs and CDs. There were significant differences in IC expression profiles of T cells from HDs versus CDs. PD-1 was expressed at significantly lower levels on the surface of CD4^+^ T cells from CDs compared with HDs basally (*p* = 0.02) (Fig. 2**B.**). TIM-3 was expressed at significantly higher levels on the surface of CD4^+^ and CD8^+^ T cells T cells from CDs compared with HDs (*p* = 0.0007 and *p* = 0.0007) (Fig. 2**D.**). LAG-3 and A2aR were expressed at significantly higher levels on the surface of CD8^+^ T cells from CDs compared with HDs (*p* = 0.004 and *p* = 0.05) (Fig. 2**E and F.**).

Intriguingly, FLOT and CROSS CTs significantly altered IC expression on the surface of T cells from both CDs and HDs (Fig. 2). Fig. 2M and 2N showcases representative flow cytometry dot plots for the specific ICs that were significantly altered post-FLOT and -CROSS CT treatment on the surface of CD4^+^ T cells (Fig. 2**M. –** PD-1, CTLA-4, KLRG-1, PD-L1 and TIM-3) and CD8^+^ T cells (Fig. 2**N. –** A2aR, KLRG-1, CTLA-4 and PD-L1) from CDs. FLOT and CROSS CT upregulated PD-1 on the surface of CD8^+^ T cells from CDs (*p* = 0.03) (Fig. 2**B.**). In contrast, CROSS CT significantly decreased TIM-3 expression on the surface of CD8^+^ T cells from CDs compared with the vehicle control (*p* = 0.01) (Fig. 2**D.**). Interestingly, FLOT significantly upregulated LAG-3 on the surface of CD4^+^ T cells from HDs and from CDs (*p* = 0.03 and *p* = 0.01) (Fig. 2**E.**). Similarly, CROSS CT significantly upregulated LAG-3 on the surface of CD4^+^ T cells from HDs compared with the vehicle control (*p* = 0.03) (Fig. 2**E.**). In contrast, CROSS CT significantly downregulated LAG-3 on the surface of CD4^+^ T cells and CD8^+^ T cells from CDs compared with the vehicle control (*p* = 0.03 and *p* = 0.04) (Fig. 2**E.**). Additionally, FLOT and CROSS CT significantly upregulated A2aR on the surface of CD4^+^ T cells and CD8^+^ T cells compared with the vehicle control from CDs (*p* = 0.02 and *p* = 0.01) (Fig. 2**F.**). FLOT and CROSS CT increased the expression of CTLA-4 on the surface of CD4^+^ T cells compared with the vehicle from HDs (*p* = 0.06 and *p* = 0.06) (Fig. 2**G.**). FLOT significantly increased the expression of CTLA-4 on the surface of CD8^+^ T cells compared with the vehicle from HDs (*p* = 0.03) and increased CTLA-4 on the surface of CD8^+^ T cells compared with the vehicle control from CDs (*p* = 0.06) (Fig. 2**G.**). FLOT and CROSS CT significantly upregulated KLRG-1 on the surface of CD4^+^ T cells compared with the vehicle control in both HDs (*p* = 0.03 and *p* = 0.03) and CDs (*p* = 0.007 and *p* = 0.007) (Fig. 2**H.**). Similarly trends were observed in the CD8 T cell compartment (Fig. 2**H.**).

FLOT significantly upregulated PD-L1 on the surface of CD4^+^ compared with the vehicle control from HDs (*p* = 0.03), and from CDs (*p* = 0.02) (Fig. 2**I.**). Similar trends were observed in the CD8^+^ T cell compartment. In addition, CROSS CT significantly upregulated PD-L1 on the surface of CD8^+^ compared with the vehicle control from CDs (*p* = 0.03) (Fig. 2**I.**). CROSS CT significantly upregulated PD-L2 on the surface of CD8^+^ T cells compared with the vehicle control from CDs (*p* = 0.05) (Fig. 2**J.**). In addition, FLOT significantly upregulated CD160 on the surface of CD4^+^ T cells compared with the vehicle control from CDs (*p* = 0.05) (Fig. 2**K.**). Similar trends were observed in the CD8^+^ T cell compartment. A heat map providing a visual summary detailing the effects of FLOT and CROSS CT on IC expression profiles of T cells from HDs versus CDs is highlighted in Fig. 2**O.** which depicts the chemotherapy-induced fold change in IC expression relative to the vehicle control. Visually it appears that 48 h chemotherapy treatment upregulated ICs on the surface of T cells derived from CDs compared with HDs Fig. 2**O.**

Overall, PD-1 was expressed at significantly lower levels on T cells from CDs compared with HDs. However, TIM-3, LAG-3 and A2aR were expressed at significantly higher levels on the surface of T cells from CDs compared with HDs. A range of ICs were directly upregulated following FLOT and CROSS CT treatment which included: PD-1, A2aR, CTLA-4, KLRG-1, PD-L1, PD-L2 and CD160. Interestingly, CROSS CT significantly decreases TIM-3 and LAG-3 on the surface of CD8^+^ T cells from CDs. FLOT and CROSS CT had a more substantial effect in altering IC expression on T cells from CDs than HDs.

**FLOT and CROSS CT regimens significantly alter the expression of co-stimulatory molecules on the surface of T cells and increase the percentage of effector memory T cells**

**Fig. 3:**
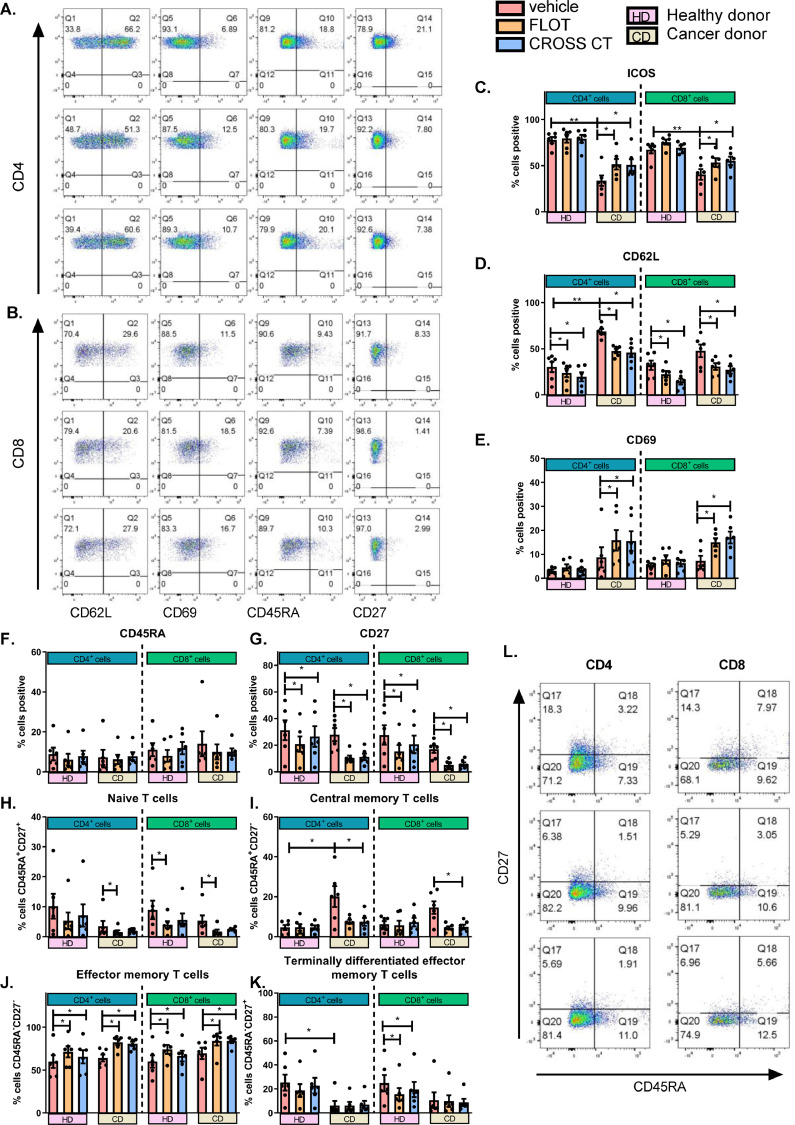
**FLOT and CROSS CT regimens upregulate co-stimulatory markers ICOS and CD69 and downregulate co-stimulatory marker CD27 on the surface of OAC T cells.** PBMCs were activated with plate bound anti-CD3 and anti-CD28 for 72 h followed by 48 h treatment with FLOT and CROSS CT regimens and expression of a range of markers reflective of T cell activation status was assessed by flow cytometry. Healthy age-matched healthy donor (HDs) PBMCs (*n* = 6) and treatment-naïve OAC cancer donor (CDs) PBMCs (*n* = 8). Representative dot plots shown in (A) demonstrating effect of vehicle, FLOT and CROSS CT on activation marker expression on CD4 (A) and CD8 cells (B) from CDs. Graphs depicting effects on activation makers ICOS (C), CD62L (D), CD69 (E), CD45RA (F) and CD27 (G). The percentage of viable naïve (CD27^+^CD45RA^+^) (H), central memory (CD27^+^CD45RA^−^) (I), effector memory (CD27^−^CD45RA^−^) (J) and terminally differentiated effector memory (CD27^−^CD45RA^+^) (K) CD4^+^ and CD8^+^ cells was also determined by flow cytometry. Representative dot plots showing effect of treatments on CD4 and CD8 T cell differentiation states are shown in (L) from CDs. *p < 0.05, **p < 0.01 and ***p < 0.001 Wilcoxon test to compare effect of treatments within HDs and CDs, and Mann Whitney to compare between HDs vs. CDs.

ICOS, which is a marker of T cell activation has been shown to play an important role in promoting effector T cell function in anti-tumour immune responses [25]. Therefore, to further interrogate the direct effects of first-line chemotherapy regimens on T cell activation status, HD and CD PBMCs were treated with either a vehicle, FLOT or CROSS CT regimens and the expression of T cell activation markers and differentiation state were subsequently profiled (Fig. 3**.**). This will help us understand if first-line chemotherapy regimens might promote or hinder T cell activation.

ICOS was expressed at significantly lower levels on the surface of T cells from CDs compared with HDs within the CD4^+^ T cell compartment (*p* = 0.002) and CD8^+^ T cell compartment (*p* = 0.008) (Fig. 3**C.**). There was also a significantly higher frequency of central memory CD4^+^ T cells from CDs compared with HDs (*p* = 0.02). CD62L was expressed at significantly higher levels on the surface of CD4^+^ T cells from CDs compared with CD4^+^ T cells from HDs (*p* = 0.002) (Fig. 3**D.**). Additionally, there was significantly lower frequencies of terminally differentiated effector memory CD4^+^ T cells from CDs compared with HDs (*p* = 0.03 and *p* = 0.02) (Fig. 3**K.**).

FLOT and CROSS CT significantly altered the expression of T cell activation markers in HDs and CDs. Interestingly, FLOT and CROSS CT significantly increased ICOS expression on the surface of CD4^+^ T cells (*p* = 0.03 and *p* = 0.03) and CD8^+^ T cells (*p* = 0.03 and *p* = 0.03) compared with the vehicle control from CDs (Fig. 3**C.**). FLOT and CROSS CT significantly decreased CD62L expression on the surface of CD4^+^ T cells compared with the vehicle control in both HDs (*p* = 0.03 and *p* = 0.03) and CDs (*p* = 0.03 and *p* = 0.03) (Fig. 3**D.**). Similarly, FLOT and CROSS CT also significantly decreased CD62L expression on the surface of CD8^+^ T cells compared with the vehicle control in both HDs (*p* = 0.03 and *p* = 0.03) and CDs (*p* = 0.03 and *p* = 0.03) (Fig. 3**D.**). FLOT and CROSS CT significantly increased CD69 expression on the surface of CD4^+^ T cells (*p* = 0.03 and *p* = 0.03) and CD8^+^ T cells compared with the vehicle control from CDs (*p* = 0.03 and *p* = 0.03) but not HDs (Fig. 3**E.**). In contrast, FLOT and CROSS CT significantly decreased CD27 expression on the surface of CD4^+^ T cells compared with the vehicle control in both HDs (*p* = 0.03 and *p* = 0.03) and CDs (*p* = 0.03 and *p* = 0.03) (Fig. 3**G.**). FLOT and CROSS CT significantly decreased CD27 expression on the surface of CD8^+^ T cells compared with the vehicle control in both HDs (*p* = 0.03 and *p* = 0.03) and CDs (*p* = 0.03 and *p* = 0.03) (Fig. 3**G.**). Chemotherapy treatment had no significant effects on CD45RA expression on the surface of T cells in both HDs and CDs (Fig. 3**F.**).

FLOT significantly decreased the percentage of naïve CD4^+^ T cells compared with the vehicle control in CDs (*p* = 0.03) (Fig. 3**H.**). However, FLOT significantly decreased the percentage of naïve CD8^+^ T cells compared with the vehicle control in both CDs (*p* = 0.03) and HDs (*p* = 0.03) (Fig. 3**H.**). The percentage of central memory CD4^+^ T cells was significantly decreased post-CROSS CT treatment compared with the vehicle control in CDs (*p* = 0.03) but not HDs (Fig. 3**I.**). Similar trends were observed whereby CROSS CT significantly decreased the frequency of central memory CD8^+^T cells compared with the vehicle control in CDs (*p* = 0.03) (Fig. 3**I.**). FLOT and CROSS CT significantly increased the frequency of effector memory CD4^+^ T cells compared with the vehicle control in CDs (*p* = 0.03 and *p* = 0.03) and HDs (*p* = 0.03 and *p* = 0.03) (Fig. 3**J.**). Similarly, FLOT and CROSS CT significantly increased the frequency of effector memory CD8^+^ T cells compared with the vehicle control in CDs (*p* = 0.03 and *p* = 0.03) and HDs (*p* = 0.03 and *p* = 0.03) (Fig. 3**J.**). FLOT and CROSS CT significantly decreased the percentage of terminally differentiated effector memory CD8^+^ T cells compared with the vehicle control in HDs (*p* = 0.03 and *p* = 0.03) (Fig. 3**K.**).

To conclude, T cells from CDs expressed significantly lower levels of co-stimulatory IC ICOS on their surface compared with HDs. Furthermore, first-line combination chemotherapy regimens substantially altered the expression of co-stimulatory ICs on the surface of T cells from CDs and HDs, as well as T cell differentiation status. Both FLOT and CROSS CT significantly reduced the expression of co-stimulatory IC CD27 on the surface T cells from both CDs and HDs, whereas FLOT and CROSS significantly upregulated co-stimulatory IC CD69 on the surface of CD T cells but not HD T cells. In addition, FLOT and CROSS CT significantly increased the frequency of effector memory T cells in both CD- and HD-derived PBMCs.

**FLOT and CROSS CT regimens enhance the production of pro-inflammatory cytokines IFN-γ and TNF-α and decrease IL-2 production in OAC T cells *ex vivo***

**Fig. 4:**
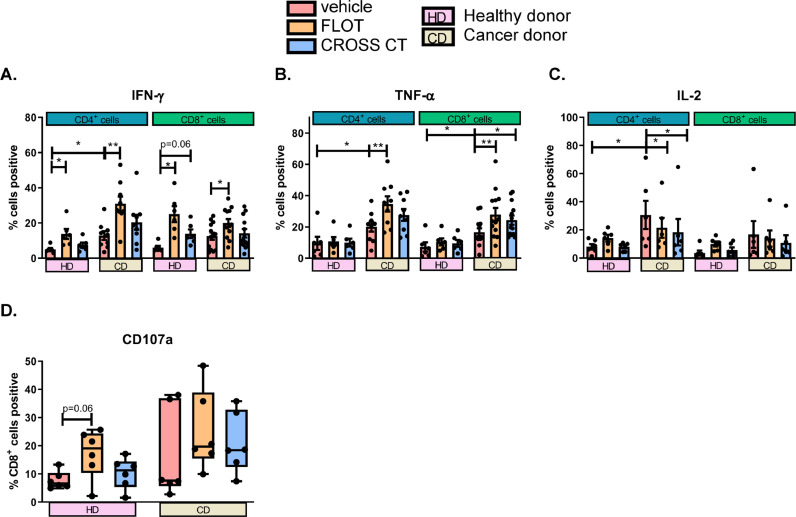
**FLOT and CROSS CT regimens increase the percentage of TNF-α and IFN-γ producing OAC T cells and decrease the percentage of IL-2 producing OAC T cells *ex vivo.*** Age-matched healthy donor (HDs) (*n* = 6) and OAC donor (CDs) (*n* = 6) PBMCs were activated with plate bound anti-CD3 and anti-CD28 for 72 h followed by 48 h treatment with FLOT and CROSS CT regimens or vehicle control (veh). The percentage of CD4^+^ and CD8^+^ cells producing IFN-γ (A), TNF-α (B) and IL-2 (C) were assessed by intracellular flow cytometry. The percentage of degranulating CD8^+^ T cells was also determined using CD107a degranulation by flow cytometry (D). *p < 0.05, **p < 0.01 Wilcoxon test to compare effect of treatments within HDs and CDs, and Mann Whitney to compare between HDs vs. CDs.

To acquire a deeper understanding of the direct immunostimulatory or immunoinhibitory effects of first-line chemotherapy regimens on T cells, activated HD- and CD-derived PBMCs were treated with either a vehicle, FLOT or CROSS CT regimens and production of anti-tumour cytokines and the cytotoxic potential of T cells was assessed (Fig. 4**.**). These experiments will help decipher whether first-line chemotherapy regimens is having an effect on anti-tumour cytokine profiles in terms of either promoting or dampening production of important anti-tumour effector functions.

Circulating CD4^+^ T cells from CDs produced significantly higher amounts of IFN-γ compared with CD4^+^ T cells from HDs (*p* = 0.03) (Fig. 4**A.**). In addition, FLOT significantly increased IFN-γ production compared with the vehicle control in CD4^+^ T cells from both CDs and HDs (*p* = 0.008 and *p* = 0.03) (Fig. 4**A.**) Similarly, FLOT significantly increased IFN-γ production compared with the vehicle control in CD8^+^ T cells from both CDs and HDs (*p* = 0.03 and *p* = 0.01) (Fig. 4**A.**)

Furthermore, CD4^+^ T cells from CDs produced significantly higher amounts of TNF-α compared with CD4^+^ T cells from HDs (*p* = 0.04) (Fig. 4**B.**). Similar trends were found within the CD8^+^ T cell compartment, where CD8^+^ T cells from CDs produced significantly higher amounts of TNF-α compared with CD8^+^ T cells from HDs (*p* = 0.02) (Fig. 4**B.**). Interestingly, FLOT significantly increased the production of TNF-α by CD4^+^ T cells and CD8^+^ T cells compared with the vehicle control from CDs but not from HDs (*p* = 0.003 and *p* = 0.002) (Fig. 4**B.**). Similarly, CROSS CT significantly increased the production of TNF-α by CD8^+^ T cells compared with the vehicle control from CDs but not from HDs (*p* = 0.02) (Fig. 4**B.**).

CD4^+^ T cells from CDs produced significantly higher amounts of IL-2 compared with CD4^+^ T cells from HDs (*p* = 0.04) (Fig. 4**C.**). In addition, FLOT and CROSS CT significantly decreased the production of IL-2 by CD4^+^ T cells compared with the vehicle control in CDs (*p* = 0.03 and *p* = 0.03) (Fig. 4**C.**). There was an increase in CD107a degranulation post-FLOT compared with the vehicle control in (*p* = 0.06) (Fig. 4**D.**).

Overall, chemotherapy treatment had a more substantial effect in altering T cell cytokine profiles from CDs compared with HDs. Chemotherapy treatment significantly increased the production of pro-inflammatory IFN-γ and TNF-α cytokines and significantly decreased IL-2 production in T cells from CDs.

**Blockade of the PD-1 signalling axis decreases LAG-3, CTLA-4 and PD-L1 and increases PD-L2 on the surface of OAC T cells *ex vivo***

Koyama et al., demonstrated that TIM-3 upregulation following PD-1 blockade was a mechanism of acquired resistance to nivolumab in non-small cell lung cancer patients [26]. This study investigated if nivolumab, atezolizumab, A2aR antagonism, dual nivolumab-atezolizumab or dual nivolumab-A2aR antagonism affected the expression of ICs on the surface of T cells in the context of OAC, which might contribute to the development of ICB resistance in OAC patients. The primary research question here was to determine if ICB might upregulate additional ICs that could be a contributing factor in the development of acquired resistance to ICB.

**Fig. 5:**
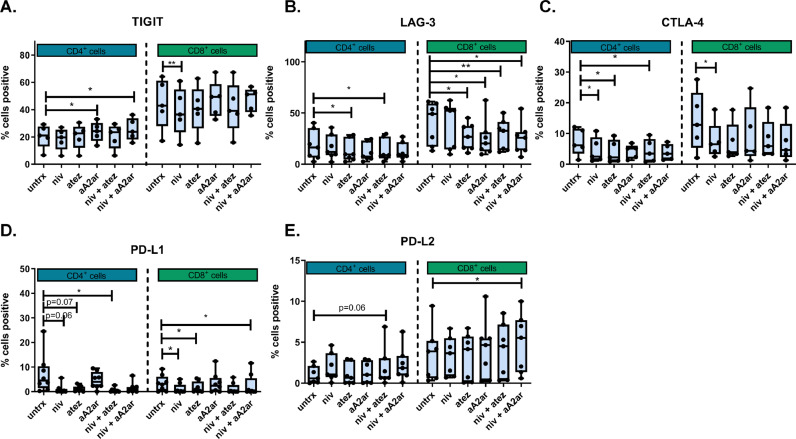
**Blockade of the PD-1 signalling axis decreases the percentage of LAG-3^+^, CTLA-4^+^ and PD-L1^+^ T cells, whereas dual nivolumab-a2aR antagonism increases the percentage of PD-L2^+^T cells *ex vivo*.** OAC donor PBMCs were activated with plate bound anti-CD3 and anti-CD28 and IL-2 for 72 h followed by 48 h treatment single agent nivolumab, atezolizumab, A2aR antagonist, dual nivolumab-atezolizumab and dual nivolumab-A2aR antagonist. The percentage of viable CD4^+^ and CD8^+^ cells expressing IC receptors (PD-1, TIGIT, TIM-3, LAG-3, A2aR, CTLA-4 and KLRG1) and IC ligands (PD-L1, PD-L2 and CD160) was assessed by flow cytometry (*n* = 8). Only data showing effect of ICBs on TIGIT (A), LAG-3 (B), CTLA-4 (C), PD-L1 (D) and PD-L2 (E) expression shown. *p < 0.05, **p < 0.01, Benjamini-Hochberg.

A2aR antagonism and dual nivolumab-A2aR antagonism significantly upregulated TIGIT on the surface of CD4^+^ T cells compared with untreated cells (*p* = 0.03 and *p* = 0.02) (Fig. 5**A.**). In contrast nivolumab significantly decreased the expression of TIGIT on the surface of CD8^+^ T cells compared with untreated cells (*p* = 0.009) (Fig. 5**A.**).

Atezolizumab and dual nivolumab-atezolizumab significantly decreased LAG-3 expression on the surface of CD4^+^ T cells compared with untreated cells (*p* = 0.03 and *p* = 0.03) (Fig. 5**B.**). Similarly, atezolizumab, A2aR antagonism, dual nivolumab-atezolizumab and dual nivolumab-A2aR antagonism significantly decreased LAG-3 expression on the surface of CD8^+^ T cells compared with untreated cells (*p* = 0.01, *p* = 0.03, *p* = 0.007 and *p* = 0.01) (Fig. 5**B.**).

Nivolumab, atezolizumab and dual nivolumab-atezolizumab significantly decreased CTLA-4 expression on the surface of CD4^+^ T cells compared with untreated cells (*p* = 0.03, *p* = 0.01 and *p* = 0.02) (Fig. 5**C.**). Similarly, nivolumab significantly decreased CTLA-4 expression on the surface of CD8^+^ T cells compared with untreated cells (*p* = 0.04) (Fig. 5**C.**).

Furthermore, nivolumab and dual nivolumab-A2aR antagonism significantly decreased PD-L1 expression on the surface of CD8^+^ T cells compared with untreated cells (*p* = 0.01 and *p* = 0.01) (Fig. 5**D.**). Dual nivolumab-A2aR antagonism significantly increased the expression of PD-L2 on the surface of CD8^+^ T cells compared with untreated cells (*p* = 0.02) (Fig. 5**E.**).

Overall, ICB significantly altered IC expression on the surface of OAC T cells increasing T cell expression of TIGIT and PD-L2 and decreasing T cell expression of LAG-3, CTLA-4 and PD-L1.

**Single and combination blockade of the PD-1, PD-L1 and A2aR pathways attenuates the FLOT- and CROSS CT-induced CD27 downregulation on the surface of OAC T cells and promotes differentiation of effector memory T cells toward a terminally differentiated state**

To further understand if ICB might synergise with chemotherapy treatment in OAC we investigated what effect combining ICB with FLOT or CROSS CT regimens has the activation status of OAC T cells *ex vivo.* We had previously shown above that chemotherapy downregulated CD27 co-stimulatory marker an important signalling molecule in promoting T cell activation and proliferation, therefore we sought to investigate whether ICB might attenuate these undesirable effects of chemotherapy on T cell activation. For these set of experiments, we pre-activated OAC-derived PBMCs for 2 days using a T cell activation protocol and then treated these T cells with ICB including nivolumab, atezolizumab or A2aR antagonist or a combination of these ICBs with and without chemotherapy. Given that PD-L1, PD-1 and A2aR are thought to primarily regulate effector T cell function in the periphery post initial T cell activation (which is in contrast to CTLA-4 which regulates initial T cell activation in the lymph node), we set up an experimental design to test whether targeting these ICs (PD-L1, PD-1 or A2aR) might propagate T cell activation in the absence and presence of chemotherapy post initial T cell priming. For this reason, we did not include ipilimumab in these experiments as CTLA-4 primarily regulates initial T cell priming.

Single agent nivolumab and atezolizumab or dual nivolumab-A2aR antagonism significantly increased CD27 expression on the surface of CD4^+^ T cells ex vivo compared with the vehicle control (*p* = 0.03, *p* = 0.03, *p* = 0.03) (Fig. 6**A.**). Similarly, there were trends toward an increase in CD27 expression on the surface of CD4^+^ T cells following single agent A2aR antagonism compared with the vehicle control (*p* = 0.06) (Fig. 6**A.**).

**Fig.. 6:**
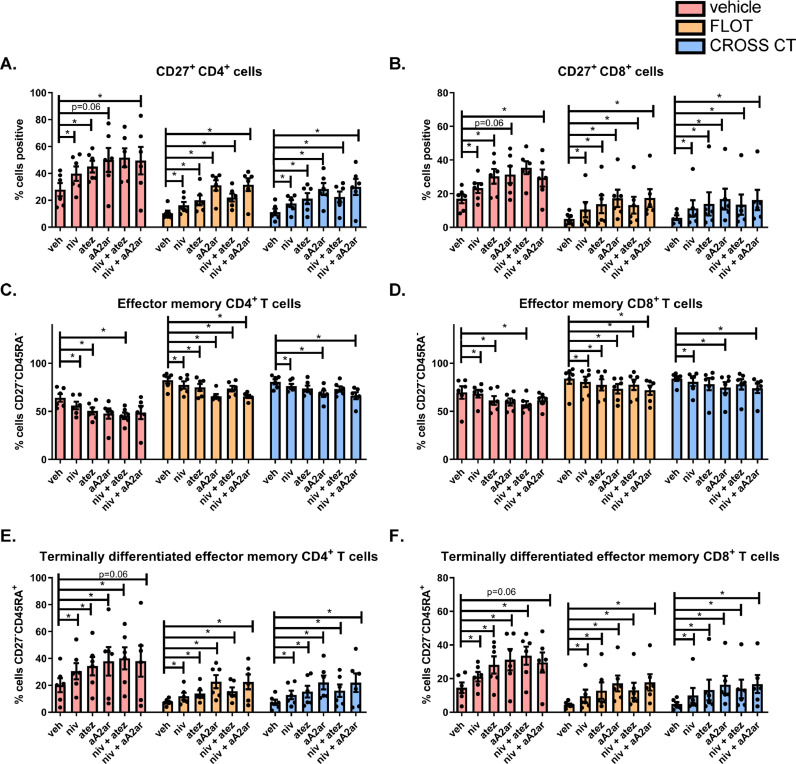
**Single agent nivolumab, atezolizumab, A2aR antagonism and dual immune checkpoint blockade attenuates chemotherapy-induced downregulation of CD27 on the surface of T cells and promotes differentiation of effector memory cells into a terminally differentiated state *ex vivo*.** OAC donor PBMCs (*n* = 8) were activated with plate bound anti-CD3 and anti-CD28 for 72 h followed by 48 h treatment with FLOT and CROSS CT regimens in the absence or presence of single agent nivolumab, atezolizumab, A2aR antagonist, dual nivolumab-atezolizumab, dual nivolumab-A2aR antagonist or vehicle control (veh). Expression of a range of markers reflective of T cell activation status (ICOS, CD27, CD69, CD62L and CD45RA) was assessed on viable CD4^+^ and CD8^+^ cells by flow cytometry. Only effect on CD27 expression on CD4 (A) and CD8 (B) cells is shown. The effect on T cell differentiation states was assessed including percentage of viable naïve (CD27^+^CD45RA^+^), central memory (CD27^+^CD45RA^−^), effector memory (CD27^−^CD45RA^−^) and terminally differentiated effector memory (CD27^−^CD45RA^+^) CD3^+^CD4^+^ and CD3^+^CD8^+^ cells by flow cytometry. Only effect on effector memory and terminally differentiated effector memory CD4 (C and E) and CD8 (D and F) cells is shown. Untreated control arms also shown in Fig. 3. *p < 0.05, Benjamini-Hochberg.

Although FLOT significantly decreased CD27 expression on the surface of CD4^+^ T cells compared with the vehicle control (*p* = 0.03), the addition of ICB with concomitant FLOT treatment attenuated FLOT-induced downregulation of CD27 on the surface of CD4^+^ T cells (Fig. 6**A.**). Single agent nivolumab, atezolizumab, A2aR antagonism, dual nivolumab-atezolizumab dual and nivolumab-A2aR antagonism significantly increased CD27 on the surface of CD4^+^ T cells in combination with FLOT compared with FLOT treated cells (*p* = 0.03, *p* = 0.03, *p* = 0.03, *p* = 0.03, *p* = 0.03, *p* = 0.03) (Fig. 6**A.**). Similar trends were found within the CD8^+^T cell compartment.

CROSS CT also significantly decreased CD27 expression on the surface of CD4^+^ T cells compared with untreated cells (*p* = 0.03) however, the addition of ICB with concomitant CROSS CT treatment attenuated CROSS CT-induced downregulation of CD27 on the surface of CD4^+^ T cells (Fig. 6**A.**). Single agent nivolumab, atezolizumab, A2aR antagonism, dual nivolumab-atezolizumab dual and nivolumab-A2aR antagonism significantly increased CD27 on the surface of CD4^+^ T cells in combination with CROSS CT compared with CROSS CT only treated cells (*p* = 0.03, *p* = 0.03, *p* = 0.03, *p* = 0.03, *p* = 0.03, *p* = 0.03) (Fig. 6**A.**). Similar trends were found within the CD8^+^T cell compartment.

Single agent nivolumab, atezolizumab and dual nivolumab-atezolizumab significantly decreased the frequency of effector memory CD4^+^ T cells compared with the vehicle control (*p* = 0.03, *p* = 0.03, *p* = 0.03) (Fig. 6**C.**).Single agent nivolumab, atezolizumab, A2aR antagonism or dual nivolumab-atezolizumab significantly increased the frequency of terminally differentiated effector memory CD4^+^ T cells compared with the vehicle control (*p* = 0.03, *p* = 0.03, *p* = 0.03, *p* = 0.03) (Fig. 6**C.**).

Although FLOT significantly increased the frequency of effector memory CD4^+^ T cells compared with the vehicle control (*p* = 0.03) and subsequently decreased the frequency of terminally differentiated effector memory CD4^+^ T cells compared (*p* = 0.06), the addition of ICB attenuated these effects (Fig. 6**C.**). ICB decreased the frequency of effector memory CD4^+^ T cells and subsequently increased the frequency of terminally differentiated CD4^+^ memory T cells with concomitant FLOT treatment (Fig. 6**E.**).

Single agent nivolumab, atezolizumab, A2aR antagonism, dual nivolumab-atezolizumab and dual nivolumab-A2aR antagonism significantly decreased the frequency of effector memory CD4^+^ T cells in combination with FLOT compared with FLOT treated cells (*p* = 0.03, *p* = 0.03, *p* = 0.03, *p* = 0.03 and *p* = 0.03) (Fig. 6**C.**). Single agent nivolumab, atezolizumab, A2aR antagonism or dual nivolumab-atezolizumab in combination with FLOT significantly increased the frequency of terminally differentiated effector memory CD4^+^ T cells compared with FLOT treated cells (*p* = 0.03, *p* = 0.03, *p* = 0.03, *p* = 0.03 and *p* = 0.03) (Fig. 6**E.**). Similar effects were observed within the CD8^+^ T cell compartment) (Fig. 6**B and D.**).

CROSS CT significantly increased the frequency of effector memory CD4^+^ T cells compared with the vehicle control (*p* = 0.03) and subsequently decreased the frequency of terminally differentiated effector memory CD4^+^ T cells (*p* = 0.03), the addition of ICB attenuated these effects (Fig. 6**C and E.**). ICB decreased the frequency of effector memory CD4^+^ T cells and subsequently increased the frequency of terminally differentiated CD4^+^ memory T cells with concomitant CROSS CT treatment (Fig. 6**C and E.**).

Single agent nivolumab, A2aR antagonism and dual nivolumab-A2aR antagonism significantly decreased the frequency of effector memory CD4^+^ T cells in combination with CROSS CT compared with CROSS CT treated cells (*p* = 0.03, *p* = 0.03, *p* = 0.03, *p* = 0.03) (Fig. 6**C.**). Subsequently, single agent nivolumab, atezolizumab, A2aR antagonism, dual nivolumab-atezolizumab, or dual nivolumab-A2aR antagonism in combination with CROSS CT significantly increased the frequency of terminally differentiated effector memory CD4^+^ T cells compared with CROSS CT treated cells (*p* = 0.03, *p* = 0.03, *p* = 0.03, *p* = 0.03) (Fig. 6**E.**). Similar trends were observed within the CD8^+^ T cell compartment (Fig. 6**D and F.**).

In summary, FLOT and CROSS CT significantly decreased CD27 expression on the surface of T cells however, the addition of ICB with concomitant FLOT or CROSS CT treatment attenuated the chemotherapy-induced downregulation of CD27 on the surface of T cells*.* FLOT and CROSS CT significantly increased the frequency of effector memory T cells and decreased the frequency of terminally differentiated effector memory T cells, however, addition of ICB significantly decreased the frequency of effector memory T cells and subsequently increased the frequency of terminally differentiated effector memory T cells.

**ICB enhances lymphocyte-mediated cytolysis of OE33 cells in the presence of post-FLOT and post-CROSS CT tumour cell secretome**

**Fig. 7:**
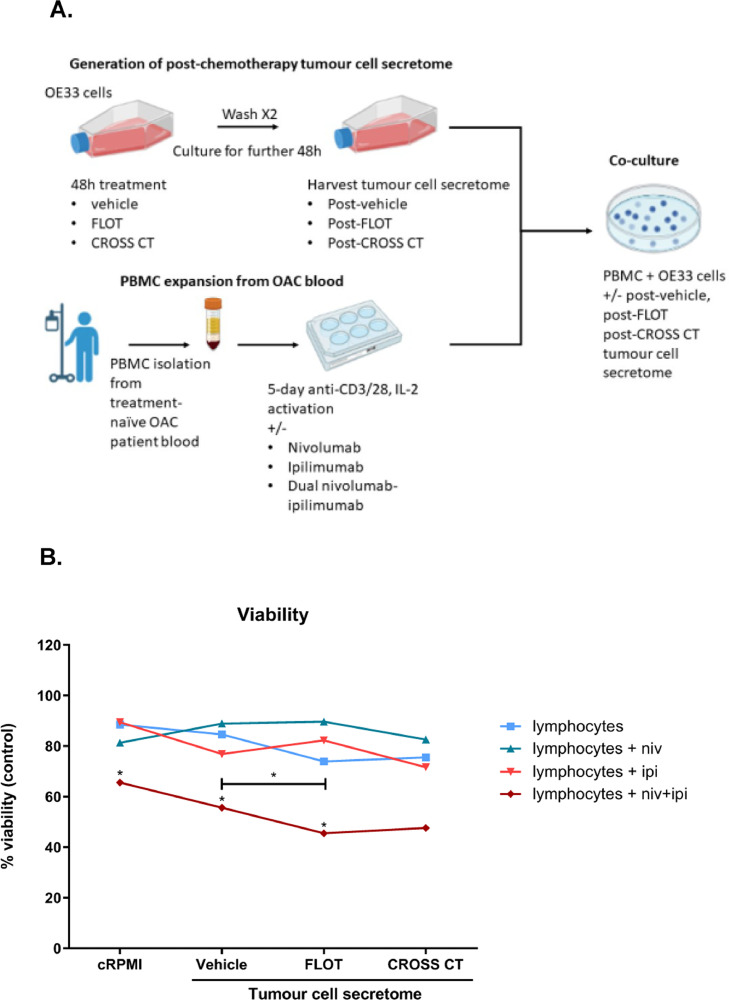
**Dual nivolumab-ipilimumab synergises with chemotherapy enhancing OAC lymphocyte-mediated killing of OE33 cells.** (A) Schematic representation of co-culture experiment. (B) OE33 cells were untreated (untrx) or treated with vehicle (veh)-, FLOT- or CROSS-treated OE33 conditioned media for 48 h. Activated treatment-naïve OAC patient-derived PBMCs were also co-cultured with OE33s in an effector:target (E:T) ratio of 5:1 (50,000:10,000) for 48 h. PBMCs pre-activated for 5 days using anti-CD3/28 in the absence or presence of nivolumab (10 μg/ml), ipilimumab (10 μg/ml) or dual nivolumab-ipilimumab (10 μg/ml and 10 μg/ml). A CCK8 assay was used to determine the viability of OE33 cells (experiments carried out *n* = 6 independent experimental repeats with duplicate technical replicates). Wilcoxon t-test. *p < 0.05 denotes a significant difference in tumour cell viability between untreated lymphocytes (blue line) versus lymphocytes treated with nivolumab (turquoise line), ipilimumab (orange line) or dual niv+ipi (red line).

In light of the findings from this study, our next research question involved investigating if ICB could enhance lymphocyte-mediated killing of tumour cells and further interrogate whether the effects of ICB could be propagated via the use of clinically relevant chemotherapy regimens (Fig. 7). To maximise the potential synergy we selected two ICBs to test; nivolumab which targets PD-1 and ipilimumab which targets CTLA-4. The rationale for selecting ipilimumab was due to the fact that ipilimumab targets CTLA-4, a non-redundant IC pathway that is thought to inhibit T cell priming in the lymph node whereas the PD-1 axis inhibits effector T cell function in peripheral organs post-initial T cell priming. In addition, the 5-day pre-activation of T cells *ex vivo* before their subsequent co-culture with OAC cells was performed to mimic the initial activation and priming of T cells in the lymph node where CTLA-4 is believed to primarily act before T cells travel to the tumour 5–7 days later to eliminate the tumour cells.

Dual nivolumab-ipilimumab treatment significantly enhanced lymphocyte-mediated killing of OE33 cells compared with untreated lymphocytes (*p* = 0.01). In addition, given that CHECKMATE 649 demonstrated that the addition of nivolumab to first-line chemotherapy regimens (FLOT/XELOX) increased the OS of oesophagogastric patients [13], we also investigated if ICB might synergise with chemotherapy treatment to enhance lymphocyte-mediated killing of OAC cells. Therefore, untreated lymphocytes and ICB-treated lymphocytes were co-cultured with OE33 cells in the absence or presence of vehicle control, FLOT- or CROSS CT-treated OE33 tumour cell secretome (Fig. 7**B.**). This experimental setup was designed to mimic the clinical scheduling of ICB with chemotherapy, as ICB is typically administered following chemotherapy (days to weeks) at a timepoint in which the tumour has been exposed and altered by the chemotherapy, but the chemotherapy has been excreted by the body (Fig. 7**A.**). In both the absence (cRPMI) and presence of vehicle-treated tumour cell secretome dual nivolumab-ipilimumab treated lymphocytes achieved the greatest level of tumour cell killing compared with lymphocytes that were not treated with ICB (Fig. 7**B.**). Interestingly, there was an even greater increase in tumour cell killing using dual nivolumab-ipilimumab treated lymphocytes in the presence of post-FLOT tumour cell secretome compared with post-vehicle tumour cell secretome (Fig. 7**B.**).

## Discussion

Chemotherapy has garnered increasing attention as a complementary partner to combine with ICB therapy to improve response rates [1]. Until recently PD-1 ICB was mainly assessed in the adjuvant setting for OAC patients as a second- or third-line agent and mainly administered as a single agent demonstrating an improvement in some but not all survival endpoints [27]. Theoretically administering ICB in the neoadjuvant setting when there is an intact and larger tumour (greater amount of tumour antigens) may have a greater probability of achieving long-term immunologic memory and tumour control as opposed to treatment in the adjuvant setting, which typically comprises of patients with micrometastatic disease and a more treatment resistant tumour [28]. Findings from the CheckMate 649 trial offer increasing support for administering ICB in the neoadjuvant setting, demonstrating that combining nivolumab with first-line chemotherapy regimens improved overall survival of OAC patients more significantly than either modality alone [13]. As previously shown in other cancer types, immunostimulatory chemotherapies synergise with ICB in ‘cold’ non-inflamed tumours by inducing immunogenic tumour cell death, mobilising tumour-specific T cells to the tumour and is often characterised by an inflamed signature [29]. This study highlighted the synergism between first-line chemotherapies used in OAC and ICB, whereby ICB was more effective in enhancing lymphocyte-mediated killing of OAC cells in the presence of post-FLOT and post-CROSS chemotherapy tumour cell secretome. The observed synergism may be due to the release of tumour antigens and DAMPs from tumour cells exposed to FLOT or CROSS chemotherapies, which could be enhancing lymphocyte activation and subsequent killing. Previous studies have shown that chemotherapy agents comprising the FLOT and CROSS regimens are immunostimulatory and induce the secretion of DAMPS from tumour cells; docetaxel (lung adenocarcinoma [30]), oxaliplatin (colorectal cancer [31] and lung carcinoma [32]) and 5-FU (colon carcinoma cells [33]) stimulate tumour cell secretion of HMGB1. Paclitaxel treatment resulted in the release of HMGB1 and activation of TLR-4-dependent and -independent pathways in ovarian cancer [34]. Similarly, oxaliplatin upregulated cell surface calreticulin expression in colorectal cancer [31] and murine lung carcinoma cell lines [35], while docetaxel increased calreticulin cell surface expression in breast, prostate and colorectal cancer cell lines. Here we have identified that both first-line chemotherapy regimens used in the OAC setting upregulate co-stimulatory markers ICOS and CD69 on the surface of T cells suggesting that these first-line chemotherapy regimens are immunostimulatory. Co-stimulatory CD69 regulates inflammation through T cell migration, retention in tissues, and plays an important role sustaining T cell activation, proliferation, cytolytic activity [36]. Tang *et al*., demonstrated that an increased frequency of ICOS^+^CD4 T cells is a pharmacodynamic biomarker of response to anti-CTLA-4 ICB [37]. A similar study highlighted that ICOS expression was shown to be an indicator of T cell-mediated immune responses following treatment with a STING agonist in Lewis lung carcinoma murine models [38]. Our data demonstrate that both FLOT and CROSS chemotherapy regimens also increase the production of pro-inflammatory IFN-γ and TNF-α cytokines supporting a rationale that FLOT and CROSS chemotherapy regimens are immunostimulatory and may synergise with ICB to enhance anti-tumour immune responses. These findings suggest that first-line chemotherapies might play an important role in ‘warming up’ a ‘cold’ non-inflamed tumour microenvironment, which are typically unresponsive to ICBs [39].

Tumours co-opt certain immune checkpoint pathways as a major mechanism of immune resistance [40]. In this study PD-1, TIM-3, LAG-3, CTLA-4 and PD-L2 were significantly upregulated on tumour-infiltrating T cells compared with peripheral circulating T cells in OAC patients, which might reflect a more exhausted T cell phenotype mediated by IC-intrinsic signalling in the tumour microenvironment. Similar findings were identified by Xie et al. who demonstrated that PD-1 and TIM-3 were significantly upregulated on tumour-infiltrating T cells in oesophageal cancer patients [41]. Furthermore, our findings identified that increased expression of ICs on T cells in circulation and infiltrating tumour tissue correlated with a poor response to subsequent neoadjuvant treatment and more advanced stage tumours. Complementary findings from a study by Xie et al. also demonstrated that expression of PD-1 and TIM-3 on CD4^+^ T cells were closely associated with clinic pathological features of oesophageal cancer patients [41]. In addition, Humphries et al., demonstrated that the levels of CD3, CD4, CD8, ICOS and PD-1 were individually predictive of better overall survival in OAC by immunohistochemistry [24]. Considering our findings, in conjunction with these studies, further underlines the therapeutic value for using ICB to treat OAC patients to improve clinical outcomes.

This study also investigated the effect of first-line chemotherapy regimens on T cell IC expression profiles, which is an important gap in research knowledge that needs to be addressed for guiding the scheduling of ICB with chemotherapy treatment. Several studies have shown that chemotherapies upregulate PD-L1 on the surface of tumour cells in colorectal, gastric [42], breast [43], head and neck [44] and lung cancer [45,46]. In this study, FLOT and CROSS chemotherapies induced upregulation of ICs on T cells, which are responsible for maintaining self-tolerance and modulate the duration and amplitude of physiological immune responses [40]. This data underlines the double-edged sword of immune stimulation whereby immune activation is always balanced with a parallel induction of immune inhibition [40]. Additionally, the hypothesis that FLOT and CROSS chemotherapy regimens might be stimulating anti-tumour immune responses is further underlined highlighting a link between chemotherapy and promotion of immune resistance mediated by ICs. These findings further suggest that combining ICBs with concomitant chemotherapy that specifically block the ICs that are upregulated by chemotherapies which include: PD-1, A2aR, CTLA-4, KLRG-1, PD-L1, PD-L2 and CD160 would represent attractive targets in the tumour microenvironment. The chemotherapy-induced upregulation of this wide array of ICs on the surface of T cells might be detrimental to the development and progression of an anti-tumour immune response and may also inhibit chemotherapy-induced immune stimulation.

In the clinic, dual blockade of CTLA-4 and PD-1 can achieve a more effective anti-tumour immune response in melanoma as both the CTLA-4 and PD-1 axes inhibit T cell activation and function using non-redundant mechanisms [47]. Combined use of nivolumab and ipilimumab has been FDA-approved in melanoma, MSI-H and DNA mismatch repair-deficient metastatic colorectal cancer and kidney cancer [48] but not in OAC patients. This study reaffirms the therapeutic potential for dual ICB whereby the highest levels of lymphocyte-mediated cytolysis of OAC cells was observed with dual nivolumab-ipilimumab treatment compared with either treatment alone.

Interestingly, CROSS chemotherapies significantly decreases TIM-3 and LAG-3 on the surface of CD8^+^ T cells. Anderson *et al*., have extensively reviewed the literature and reported that TIM-3 and LAG-3 have unique functions in anti-cancer immune responses [12]. Similarly, there were trends toward a significant reduction in TIM-3 and LAG-3 expression on the surface of tumour-infiltrating CD4^+^ T cells post-FLOT and post-CROSS chemoradiotherapy. TIM-3 and LAG-3 are highly expressed on Tregs at sites of tissue inflammation [12]. Therefore, the findings from this study might suggest that the chemotherapy-induced downregulation of TIM-3 and LAG-3 may reflect a reduction in the frequency of regulatory type T cells post-chemo(radio)therapy treatment.

An interesting study in OAC carried out by Gebauer et al. [49], identified LAG-3 on the surface of tumour-infiltrating lymphocytes in approximately 10% of cases, and found that its expression was a good prognostic indicator. Expression of LAG-3 positively correlated with the level of tumour-infiltrating of CD4^+^ and CD8^+^ T cells and LAG-3 expression identified patients with a longer overall survival compared with those who lacked LAG-3 expression (median overall survival 70.2 vs. 26.9 months; p = 0.046) [49]. A complementary study in oesophageal squamous cell carcinoma discovered that LAG-3 expression on tumour-infiltrating T cells also correlated with the level of tumour-infiltrating of CD4^+^ and CD8^+^ T cells [50]. Similarly, Zhang et al., found that LAG-3 expression identified patients with an increased progression-free survival compared with those who lacked LAG-3 expression [50].

Furthermore, FLOT and CROSS chemotherapies significantly decreased the production of IL-2 by T cells. IL-2 promotes the expansion of regulatory T cells [51], therefore, the FLOT and CROSS chemotherapy-induced decrease in IL-2 production by T cells may in turn decrease regulatory T cell expansion and support development of anti-tumour immunity. Studies have demonstrated that taxanes including docetaxel which forms part of the FLOT regimen and paclitaxel which forms part of the CROSS regimen depletes Treg cells in non-small cell lung cancer patients [52,53].

In conclusion, these findings demonstrate that there is a wide range of ICs expressed in OAC patients that represent potential therapeutic targets, outside of the well-known PD-1 and CTLA-4 axis, to propagate immune responses promoting tumour eradication. In particular, TIM-3 and LAG-3 were upregulated on tumour-infiltrating T cells and may represent novel targets in combination with PD-1 and/or CTLA-4 ICBs. However, an increased understanding of the specialized functions of these novel ICs in the context of OAC is necessary to design optimal combinations of ICBs to administer to OAC patients. Additionally, the potential synergy between ICB and first-line chemotherapy regimens in OAC are also highlighted. These findings also demonstrate that first-line chemotherapy regimens upregulate ICs on T cell surfaces reaffirming the rationale to administer ICBs concurrently with first-line chemotherapies in OAC to prevent potential IC-mediated suppression of chemotherapy-induced anti-tumour immunity.

## Author contributions

Maria Davern was responsible for study conceptualization, methodology, investigation, data curation, formal analysis, writing and editing the original draft. Noel E. Donlon and Andrew D. Sheppard were responsible for data curation and investigation. Fiona O’ Connell, Ross King and Conall Hayes carried out formal analysis. Noel E. Donlon, Hugo Temperley, Jenny Moore, Dara Bracken-Clarke, John V. Reynolds, Claire Donohoe, Christine Butler, Anshul Bhardwaj, and Narayanasamy Ravi provided resources and project administration. Melissa J. Conroy and Stephen G. Maher contributed to study conceptualization. Joanne Lysaght was responsible for study conceptualization, supervision of the project, funding acquisition and formal analysis. All authors were involved in the review and editing of the manuscript.zeg

## Declaration of Competing Interest

The authors declare that there is no conflict of interest that could be perceived as prejudicing the impartiality of the research reported.

## Appendix Supplementary materials

Image, application 1
