# Supplementary material for: Cooperation between chemotherapy and immune checkpoint blockade to enhance anti-tumour T cell immunity in oesophageal adenocarcinoma
Source: Transl Oncol. 2022 Mar 30;20:101406. doi: 10.1016/j.tranon.2022.101406 (PMC8976141; doi:10.1016/j.tranon.2022.101406)
Supplement: Supplementary file 1 [file mmc1.docx]

Supplemental

**Figure S1: Expression of IC receptors and ligands on the surface of T cells in the treatment-naïve setting versus post-treatment setting in OAC patients.** (A) Graphs displaying the frequency of CD4^+^ and CD8^+^ T cells expressing ICs in circulation and infiltrating tumour tissue in the treatment-naïve versus post-treatment setting (combining post-FLOT and post-CROSS CRT samples). Patient cohort includes treatment-naïve OAC patients (blood: n=17 and tumour: n=10), post-FLOT (blood: n=6 and tumour: n=6) and post-CROSS CRT (blood: n=4 and tumour: n=4). Mann Whitney test *p<0.05, **p<0.01.
